# Post-pancreatectomy Acute Pancreatitis in Distal Pancreatectomies — a Rare Bird According to the New Definition

**DOI:** 10.1007/s11605-023-05721-w

**Published:** 2023-06-12

**Authors:** Holmberg Marcus, Kartalis Nikolaos, Larsson Patrik, Swartling Oskar, Linder Stefan, Gilg Stefan, Sparrelid Ernesto, Ghorbani Poya

**Affiliations:** 1 Department of Clinical Science, Intervention and Technology, Karolinska Institute, Stockholm, Sweden; 2 Department of Surgery, Capio St Görans Hospital, Stockholm, Sweden; 3 Department of Radiology, Karolinska University Hospital, Stockholm, Sweden; 4 Department of Upper Gastrointestinal Diseases, Karolinska University Hospital, Stockholm, Sweden; 5 Division of Clinical Epidemiology, Department of Medicine, Karolinska Institute, Stockholm, Sweden

**Keywords:** PPAP, CT, Amylase, Complications

## Abstract

**Background:**

Post-pancreatectomy acute pancreatitis (PPAP) is a recently identified clinical condition characterized by sustained elevated serum amylase levels for at least 48 h post-operatively, consistent radiological findings, and relevant clinical features. The purpose of this study was to determine the frequency of PPAP after DP, to investigate the rate of major complications in patients with sustained or transiently elevated serum amylase activity, and to explore the usability of CT as a prerequisite for the diagnosis of PPAP.

**Methods:**

This retrospective single-center observational study included consecutive patients 18 years or older who underwent DP at Karolinska University Hospital between 2008 and 2020. The two serum amylase levels on post-operative days (POD) 1 and 2 were correlated with post-operative major complications by logistic regression analyses.

**Results:**

Of the 403 patients who underwent DP, 14% (*n* = 58) had sustained elevated serum amylase levels according to PPAP criteria, and 31% (*n* = 126) had transiently elevated serum amylase levels on either POD1 or POD2. Of the patients with sustained elevated levels, 45% (*n* = 26) developed major complications, but less than 2% (*n* = 1) showed imaging findings consistent with acute pancreatitis. Of the 126 patients who exhibited only transiently elevated serum amylase on either POD1 or POD2, 38% (*n* = 48) developed major complications. The frequency of PPAP was 0.25% (*n* = 1).

**Conclusion:**

These findings indicate that PPAP after DP is rare and that computed tomography has limited usability for diagnosing PPAP. The findings also suggest that transiently elevated serum amylase may be an early indicator of acute pancreatitis, especially when peaked.

## Introduction

Acute pancreatitis following distal pancreatectomy ^[1]^ may trigger post-operative complications.^[1–3]^ The International Study Group for Pancreatic Surgery recently defined post-pancreatectomy acute pancreatitis (PPAP) as a clinical entity.^[4]^

The diagnosis of PPAP is based on post-operative serum hyperamylasemia (POH) greater than the institutional upper limit for normal sustained elevated for at least the first 48 h after surgery, imaging findings consistent with acute pancreatitis, and associated clinically relevant features. The revised Atlanta classification for acute pancreatitis in non-surgical settings also utilizes biochemical, clinical, and radiologic criteria for diagnosis, but only two of these three criteria are required for diagnosis.^[5]^ PPAP may in turn result in post-operative pancreatic fistula (POPF), post-pancreatectomy hemorrhage,^[6]^ and intra-abdominal abscess/sepsis.^[2,4]^

The understanding of POH dynamics and acute pancreatitis after DP is limited. As for acute pancreatitis in non-surgical settings and after pancreatoduodenectomy,^[7]^ it is plausible that acute pancreatitis after DP may be preceded by only a transient peak of serum amylase activity. The diagnostic requirement of sustained serum amylase levels for the diagnosis of PPAP may therefore overlook significant cases with only transiently elevated serum amylase activity.

This study aims to determine the frequency of PPAP after DP, to investigate the rate of major complications in patients with sustained or transiently elevated serum amylase activity, and to explore the usability of CT as a prerequisite for the diagnosis of PPAP.

## Materials and Methods

This retrospective observational cohort study was approved by the local Ethical Committee of Stockholm (registration number: DNr 2020/05238) and is reported in accordance with the Strengthening the Reporting of Observational Studies in Epidemiology (STROBE) guidelines ^[8]^.

### Study Population

All adults patients (age ≥ 18 years) undergoing DP between 1st of January 2008 to 31st of December 2020 at Karolinska University Hospital, Stockholm, Sweden, were considered for the study (*n* = 500). Data were retrospectively collected and analyzed. Patients with missing serum amylase on POD1 and POD2 were excluded (*n* = 97). Last follow up was 31st of December 2022.

### Covariates and Definitions

Data was collected regarding surgical technique: open or minimally invasive; preservation of the spleen or not; and standard or extended resection, defined according to the ISGPS.^[9]^ Pancreatic transection was performed using either electric cautery, scalpel, or stapler. Transection to the right of the portal vein/superior mesenteric vein was referred as “intrapancreatic extended resection.” The pancreatic remnant was closed by stapling, hand sewn technique, or a combination of these methods. The pancreatic texture was graded as soft, intermediate, or hard.^[10]^ The diameter of the main pancreatic duct (≤ 3 mm or > 3 mm) was estimated on pre-operative CT or measured on the operative specimen.

Surgery typically began at 9 am. Laboratory data, including CRP levels (mg/L), serum and drain amylase activity (µ-kat/L), and white blood cell count (10^9^/L), were collected at 6 am on POD 1, 2, and 3. The use of somatostatin analogs during the perioperative period was selectively administered to patients with high-risk pancreas, particularly those with soft tissue, based on the surgeon’s discretion, and treatment was occasionally started several days after surgery.

The institutional upper limit for normal serum amylase activity was 1.15 μ-kat/L (equivalent to 69 IU/L). Serum amylase activity was classified as “normal” if it was within normal limits on both POD1 and POD2; “sustained elevated” if serum amylase activity was elevated on both POD1 and POD2, following the ISGPS PPAP criterion;^[4]^ and “transiently elevated” if it was above normal on either day. Serum amylase activity was therefore categorized into three main levels: “normal,” “transiently elevated,” and “sustained elevated.” In some analyses, serum amylase activity ≥ 3 times normal was used in conjunction with sustained and transient elevation and referred to as “peaked,” following the revised Atlanta classification.^[5]^ CRP levels were a priori chosen for POD2 (< 180 and ≥ 180 mg/L)^[2]^ and for POD3 ((< 180, 180–199 and ≥ 200 mg/L).

During the study period, there were no predetermined criteria for indicating the need for imaging. Post-operative computed tomography (CT) of the abdomen during the portal venous phase after the intravenous administration of iodine-based contrast agent was performed when deemed necessary based on the clinical course, and the threshold for performing emergency CT after DP at current institution was generally low. For the purpose of the study, only CT examinations performed within the first week were re-evaluated for imaging findings suggestive of acute pancreatitis by one radiologist (NK) with 18 years post-residency experience in pancreatic imaging.

The complications POPF, PPH, delayed gastric emptying (DGE), and PPAP were defined according to current ISGPS definitions.^[11–13]^ Post-operative complications were graded according to the Clavien–Dindo classification system^[14]^ with a cut-off at 90 days. The primary outcome to this study was major complications, defined as Clavien–Dindo grade 3a or higher. All pertinent data and outcomes were analyzed.

### Statistical Analyses

In descriptive statistics, pre-, intra-, and post-operative covariates were compared using Kruskal–Wallis rank sum test or Wilcoxon rank sum test (depending on the number of comparison groups) for continuous covariates and Pearson’s Chi-squared test (or Fisher’s exact test when appropriate) for categorical variables. Continuous covariates were presented as medians and interquartile ranges (IQR), whereas categorical variables were presented as percentages and frequencies.

Five multivariable binary logistic regression analyses were utilized. The first two analyses aimed to investigate the correlation between pre- and intra-operative covariates and the risk of developing either sustained elevated serum amylase activity on one side or transiently elevated activity ≥ 3 times the upper limit of normal on the other side. The third regression aimed to examine the association between perioperative laboratory results and major complications. The laboratory tests included serum amylase activity (measured pre-operatively and on POD1–3), PPAP criterion, Atlanta criterion, drain amylase activity (measured on POD1–3), CRP levels (measured pre-operatively and on POD2–3), and white blood cell count (measured on POD1–3).

The fourth and fifth regressions investigated the correlation between pre- and intra-operative and laboratory covariates, respectively, and the risk of developing POPF. The laboratory tests included serum amylase activity (measured on POD1-3), PPAP criterion, Atlanta criterion, drain amylase activity on POD3, CRP levels on POD2, and white blood cell count (measured on POD1-3).

To identify significant covariates, each regression analysis started with a univariable regression, and covariates that demonstrated a significant association (*p* < 0.05) were included in the subsequent multivariable logistic regression. To avoid interactions between measurement days for laboratory covariates, the most significant day for each covariate was selected for the multivariable analysis. If both PPAP and Atlanta criteria demonstrated a significant association, we planned to conduct two separate multivariable regressions; however, this was not necessary.

Backward stepwise regression was used, starting with a saturated model, to exclude variables with *p* > 0.1 at each step until no more variables could be excluded. The effect of covariates on the outcome was calculated and presented as odds ratio (OR) with 95% confidence intervals (CI). In all analyses, the level of statistical significance was set to 5%. We performed data analyses using R version 4.1.2 (Vienna, Austria. 2020).

## Results

During the study period, 403 consecutive patients underwent DP. Out of these, 58 patients showed sustained elevated serum amylase levels according to the PPAP criteria, while 126 patients had only transiently elevated serum amylase levels on either POD1 or POD2, failing to meet the PPAP criterion. The median age of all patients was 67 years, and female sex was slightly more frequent (Table 1). Two-thirds of the patients had an ASA grade of 1 or 2, and the median BMI was 26 kg/m^2^. The majority of the procedures (87%) were performed using laparotomy with 94% of them including splenectomy. Extended resection (including intrapancreatic) was necessary in one-third of the cases. The median procedure time was 201 min, with a median estimated intra-operative blood loss of 250 ml.

**Table 1:** Baseline and operative characteristics

		S-Amylase			Elevated S-amylase		
Variable	Overall *N* = 403^*1*^	Normal *n* = 219^*1*^	Elevated *n* = 184^*1*^	*p* value^*2*^	Transiently *n* = 126^*1*^	Sustained *n* = 58^*1*^	*p* value^*2*^
Sex				0.159			0.090
Female	219 (54)	112 (51)	107 (58)		68 (54)	39 (67)	
Male	184 (46)	107 (49)	77 (42)		58 (46)	19 (33)	
Age				**0.020**			0.567
< 60	122 (32)	61 (29)	61 (35)		40 (33)	21 (40)	
60–74	180 (47)	93 (44)	87 (50)		61 (50)	26 (49)	
≥ 75	82 (21)	56 (27)	26 (15)		20 (17)	6 (11)	
ASA				** < 0.001**			0.338
1 to 2	239 (63)	115 (55)	124 (73)		85 (71)	39 (78)	
3 to 4	139 (37)	93 (45)	46 (27)		35 (29)	11 (22)	
BMI				0.894			0.346
< 25	171 (45)	94 (45)	77 (44)		50 (41)	27 (51)	
25–29	137 (36)	73 (35)	64 (37)		45 (37)	19 (36)	
≥ 30	76 (20)	43 (20)	33 (19)		26 (21)	7 (13)	
Smoking	58 (14)	35 (16)	23 (13)	0.343	16 (13)	7 (12)	0.922
Neoadj. chemotherapy	12 (3.1)	5 (2.4)	7 (4.0)	0.357	6 (5.0)	1 (1.9)	0.677
Procedure				0.383			0.059
Open	333 (87)	185 (88)	148 (85)		107 (88)	41 (77)	
Minimally invasive	51 (13)	25 (12)	26 (15)		14 (12)	12 (23)	
Spleen pres	24 (6.2)	6 (2.9)	18 (10)	**0.003**	10 (8.3)	8 (15)	0.173
Procedure time				0.653			0.598
< 3 h	146 (39)	84 (40)	62 (36)		47 (39)	15 (31)	
3–4 h	105 (28)	58 (28)	47 (28)		32 (26)	15 (31)	
> 4 h	127 (34)	66 (32)	61 (36)		42 (35)	19 (39)	
Intra-operative hemorrhage				**0.041**			0.113
< 300 ml	219 (55)	131 (60)	88 (49)		64 (51)	24 (43)	
300–1000 ml	143 (36)	72 (33)	71 (39)		50 (40)	21 (38)	
> 1000 ml	37 (9.3)	15 (6.9)	22 (12)		11 (8.8)	11 (20)	
Transection				**0.035**			0.566
Stapler	186 (49)	109 (52)	77 (45)		51 (42)	26 (51)	
Suture	68 (18)	42 (20)	26 (15)		19 (16)	7 (14)	
Combination	127 (33)	58 (28)	69 (40)		51 (42)	18 (35)	
Gland texture				**0.010**			0.527
Soft	135 (33)	80 (37)	55 (30)		35 (28)	20 (34)	
Intermediate	196 (49)	92 (42)	104 (57)		72 (57)	32 (55)	
Firm	72 (18)	47 (21)	25 (14)		19 (15)	6 (10)	
Duct diameter				0.349			0.139
≤ 3 mm	242 (86)	159 (88)	83 (84)		70 (86)	13 (72)	
> 3 mm	38 (14)	22 (12)	16 (16)		11 (14)	5 (28)	
Extended resection	115 (30)	54 (26)	61 (35)	0.047	39 (32)	22 (42)	0.238
Intrapancreatic	36 (9.4)	18 (8.6)	18 (10)	0.553	12 (9.9)	6 (11)	0.780
Extrapancreatic	93 (24)	42 (20)	51 (29)	**0.034**	31 (26)	20 (38)	0.106
Vascular	17 (4.4)	6 (2.9)	11 (6.3)	0.100	6 (5.0)	5 (9.4)	0.313

^*1*^*n* (%); median (25–75%)

^*2*^Pearson’s chi-squared test; Wilcoxon rank sum test

Analysis of the patients’ laboratory characteristics in Table 2 showed that patients with elevated serum amylase activity post-operatively had higher pre-operative activity as well. Patients with sustained elevated amylase activity had higher drain amylase activity on POD 2 and 3, as well as maximum CRP levels within the first week, compared to patients with transiently elevated activity. There were no significant differences in white blood cell count between the groups. Twenty-nine patients (7.2%) had peaked serum amylase activity, 10 (34%) were transiently elevated, and 19 (66%) sustained elevated.

**Table 2:** Laboratory characteristics

		S-Amylase			Elevated S-amylase		
Variable	Overall *N* = 403^*1*^	Normal *n* = 219^*1*^	Elevated *n* = 184^*1*^	*p* value^*2*^	Transiently *n* = 126^*1*^	Sustained *n* = 58^*1*^	*p* value^*2*^
Serum amylase							
Pre-operatively	0.42 (0.28–0.62)	0.35 (0.24–0.51)	0.56 (0.38–0.82)	< 0.001	0.53 (0.37–0.75)	0.69 (0.42–0.95)	0.073
POD1	1.05 (0.61–1.63)	0.64 (0.42–0.83)	1.69 (1.40–2.45)	< 0.001	1.58 (1.36–1.96)	2.62 (1.83–3.47)	< 0.001
POD2	0.54 (0.31–0.80)	0.34 (0.24–0.49)	0.83 (0.63–1.33)	< 0.001	0.68 (0.56–0.85)	1.64 (1.34–2.20)	< 0.001
POD3	0.31 (0.19–0.54)	0.20 (0.16–0.35)	0.49 (0.32–0.97)	< 0.001	0.39 (0.29–0.57)	0.88 (0.60–1.96)	< 0.001
Drain amylase							
POD1	21.9 (9.8–42.8)	16.5 (6.7–32.4)	30.1 (12.8–54.1)	< 0.001	29.5 (12.2–48.4)	33.3 (13.8–61.8)	0.248
POD2	14.0 (4.8–39.2)	8.5 (3.2–24.3)	21.3 (9.1–62.9)	< 0.001	17.0 (8.5–43.4)	35.5 (12.3–80.5)	0.012
POD3	3.2 (1.1–13.7)	1.9 (0.7–8.7)	5.3 (2.0–26.8)	< 0.001	3.7 (1.7–15.4)	13.2 (4.6–54.5)	< 0.001
C-reactive protein							
Pre-operatively	2.0 (1.0–5.0)	2.0 (1.0–6.0)	2.0 (0.0–4.0)	0.013	2.0 (0.0–4.0)	2.0 (1.0–4.0)	0.298
POD1	43 (29–60)	41 (27–60)	44 (30–60)	0.689	43 (30–58)	46 (32–64)	0.601
POD2	106 (68–157)	103 (65–157)	114 (76–157)	0.378	112 (76–150)	116 (76–165)	0.535
POD3	162 (107–220)	153 (102–212)	172 (112–226)	0.100	168 (127–215)	186 (99–247)	0.267
Max value first week	186 (128–245)	179 (122–241)	192 (138–256)	0.044	185 (138–237)	228 (158–282)	0.048
White blood cell count							
POD1	13 (11–16)	13 (11–15)	13 (11–16)	0.458	13 (11–16)	14 (11–16)	0.385
POD2	16 (13–18)	16 (13–18)	16 (13–19)	0.378	16 (13–19)	15 (13–19)	0.559
POD3	15 (12–18)	15 (12–17)	15 (12–18)	0.100	15 (13–18)	14 (12–19)	0.694

^*1*^Median (2575%)

^*2*^Wilcoxon rank sum test

Abbreviation: *POD* post-operative day

Of all patients, one-third exhibited major morbidities post-operatively (Table 3), with POPF being the most common (33%). Almost two-thirds of the patients received antibiotics, and 25% developed a deep infection, with one in five patients requiring radiologically guided percutaneous drain placement. A CT examination within the first week was performed in 8% of patients, with only one examination showing findings consistent with acute pancreatitis.

**Table 3:** Post-operative characteristics and complications

Variable		S-Amylase			Elevated S-amylase		
	Overall *N* = 403^*1*^	Normal *n* = 219^*1*^	Elevated *n* = 184^*1*^	*p* value^*2*^	Transiently *n* = 126^*1*^	Sustained *n* = 58^*1*^	*p* value^*2*^
Major complications	144 (36)	70 (32)	74 (40)	0.085	48 (38)	26 (45)	0.387
Clavien–Dindo				0.056			0.471
0–2	259 (64)	149 (68)	110 (60)		78 (62)	32 (55)	
3	125 (31)	65 (30)	60 (33)		39 (31)	21 (36)	
4	15 (3.7)	4 (1.8)	11 (6.0)		8 (6.3)	3 (5.2)	
5	4 (1.0)	1 (0.5)	3 (1.6)		1 (0.8)	2 (3.4)	
POPF				0.139			0.011
No or A	273 (68)	153 (70)	120 (65)		85 (67)	35 (60)	
B	121 (30)	64 (29)	57 (31)		40 (32)	17 (29)	
C	9 (2.2)	2 (0.9)	7 (3.8)		1 (0.8)	6 (10)	
PPH				0.042			0.029
No or A	375 (94)	210 (96)	165 (90)		113 (90)	52 (90)	
B	18 (4.5)	5 (2.3)	13 (7.1)		11 (8.8)	2 (3.4)	
C	8 (2.0)	3 (1.4)	5 (2.7)		1 (0.8)	4 (6.9)	
DGE				> 0.999			0.374
No or A	389 (98)	212 (98)	177 (98)		122 (98)	55 (96)	
B	5 (1.3)	3 (1.4)	2 (1.1)		1 (0.8)	1 (1.8)	
C	4 (1.0)	2 (0.9)	2 (1.1)		1 (0.8)	1 (1.8)	
Drain	403 (100)	219 (100)	184 (100)	> 0.999	126 (100)	58 (100)	> 0.999
Duration (days)	8.2 (6.0–20)	8.1 (6.1–20)	8.3 (6.2–21)	0.706	9.1 (6.0–21)	8.2 (6.1–23)	0.947
Additional	75 (20)	36 (17)	39 (23)	0.176	21 (18)	18 (35)	0.014
Antibiotics	230 (60)	132 (63)	98 (57)	0.243	67 (56)	31 (60)	0.645
Duration (days)	13 (8.1–24)	13 (9.2–23)	14 (8.2–24)	0.511	14 (8.1–23)	14 (7.8–31)	0.603
Deep infection	95 (25)	52 (25)	43 (25)	0.991	28 (23)	15 (28)	0.468
Computer tomography	30 (8.1)	12 (6.1)	18 (10)	0.122	12 (10)	6 (11)	0.941
Pancreatitis	1 (3.3)	0 (0.0)	1 (5.6)	> 0.999	0 (0.0)	1 (17)	0.333
Post-operative pancreatic stent	17 (4.5)	8 (3.8)	9 (5.2)	0.502	3 (2.5)	6 (12)	0.023
Re-laparotomy	27 (6.7)	14 (6.4)	13 (7.1)	0.788	8 (6.3)	5 (8.6)	0.552
Intermediate care	324 (84)	174 (83)	150 (86)	0.368	104 (86)	46 (87)	0.882
Intensive care	16 (4.2)	5 (2.4)	11 (6.3)	0.054	7 (5.8)	4 (7.5)	0.738
Hospital stay (days)	9.2 (7.1–14)	9.1 (6.9–13)	10 (8.0–14)	0.013	10 (7.8–14)	11 (8.0–15)	0.513
Re-admission	90 (24)	50 (24)	40 (23)	0.903	24 (20)	16 (31)	0.108
Final pathology				0.514			0.146
Malignant	136 (35)	80 (38)	56 (32)		39 (32)	17 (32)	
NET	73 (19)	35 (17)	38 (22)		21 (17)	17 (32)	
Premalignant	131 (34)	71 (34)	60 (34)		46 (38)	14 (26)	
Benign	44 (11)	24 (11)	20 (11)		15 (12)	5 (9.4)	

^*1*^n (%); median (25–75%)

^*2*^Pearson’s chi-squared test; Fisher’s exact test; Wilcoxon rank sum test

Abbreviations: *POPF* post-operative pancreatic fistula; *PPH* post-operative hemorrhage; *DGE* delayed gastric emptying

Final histology showed that malignant and premalignant pathology constituted one-third each of the resections, while neuroendocrine tumors and benign pathology constituted one-fifth and one-tenth of the resections, respectively. The underlying diagnosis was not associated with post-operative serum amylase activity.

### Logistic Regressions

Independent predictors for elevated serum amylase activity were ASA grade (protective), extended extrapancreatic organ resection (adverse), and for sustained elevated also duct size ≥ 3 mm (adverse) (Table 4 part A).

**Table 4:** Logistic regressions for the outcome major complications

(A) Pre- and intra-covariates											(B) Laboratory covariates							
	Univariable				Multivariable			Multivariable				Univariable				Multivariable		
	Sustained elevated				Sustained elevated			Transiently elevated				Sustained elevated				Sustained elevated		
Characteristic	*N*	OR^*1*^	95% CI^*1*^	*p* value	OR^*1*^	95% CI^*1*^	*p* value	OR^*1*^	95% CI^*1*^	*p* value	Characteristic	*N*	OR^*1*^	95% CI^*1*^	*p* value	OR^*1*^	95% CI^*1*^	*p* value
Male sex	403	0.53	0.29, 0.94	0.035							Serum amylase							
Age	384	0.98	0.96, 1.00	0.056							Pre-operatively	332	0.96	0.76, 1.10	0.642			
ASA	378	0.44	0.27, 0.70	< 0.001	0.20	0.08, 0.48	< 0.001	0.36	0.18, 0.67	0.002	POD1	403	1.19	1.04, 1.38	0.013			
BMI	384	0.95	0.89, 1.01	0.138							POD2	403	1.15	0.97, 1.40	0.114			
Smoking	401	0.80	0.32, 1.77	0.613							POD3	380	1.11	0.85, 1.44	0.422			
NeoAdj. Ch	384	0.56	0.03, 2.97	0.582							Peaked ≥ 3 times	403						
Procedure	384										No		—	—		—	—	
Open		—	—								Yes		3.22	1.50, 7.24	0.003	3.06	1.39, 7.04	0.006
Min. inv		2.19	1.03, 4.43	0.034							Sustained elevated	403						
Spleen pres	384	3.50	1.35, 8.45	0.007							No		—	—				
Procedure time	378	1.00	1.00, 1.00	0.442							Yes		1.56	0.88, 2.74	0.120			
Hemorrhage i-op	399										Drain amylase							
< 300 ml		—	—								POD1	394	1.00	1.00, 1.01	0.089			
300–999 ml		1.40	0.74, 2.62	0.295							POD2	392	1.00	1.00, 1.01	0.205			
≥ 1000 ml		3.44	1.47, 7.73	0.003							POD3	389	1.00	1.00, 1.01	0.063			
Transection	381										CRP							
Stapler		—	—								Pre-operatively	386	1.01	0.99, 1.02	0.427			
Suture		0.71	0.27, 1.63	0.441							POD2	400						
Combination		1.02	0.52, 1.93	0.961							< 180		—	—				
Gland texture	403										≥ 180		2.09	1.23, 3.56	0.007			
Soft		—	—								POD3	397						
Intermediate		1.12	0.62, 2.09	0.710							< 180		—	—		—	—	
Firm		0.52	0.18, 1.30	0.186							180–199		1.12	0.52, 2.31	0.759	1.12	0.52, 2.33	0.759
Duct > 3 mm	280	2.67	0.81, 7.59	0.079	5.00	1.35, 17.7	0.012				≥ 200		2.40	1.53, 3.76	< 0.001	2.27	1.45, 3.59	< 0.001
Extended res	384	1.82	0.99, 3.29	0.050							Leucocytes							
Intrapancreatic	384	1.28	0.46, 3.05	0.601							POD1	396	1.05	1.01, 1.11	0.056			
Extrapancreatic	384	2.14	1.15, 3.93	0.015	3.31	1.00, 10.6	0.042	2.49	1.06, 5.70	0.032	POD2	402	1.06	1.01, 1.11	0.010			
Vascular	384	2.77	0.85, 7.83	0.066							POD3	395	1.06	1.02, 1.12	0.007			

^*1*^*OR* odds ratio, *CI* confidence interval

Independent adverse laboratory predictors for major complications were elevated serum amylase activity based on Atlanta criteria and CRP levels on POD3. In contrast, there was no significant association between laboratory PPAP criterion and major complications (Table 4 part B).

Independent pre- and intra-operative predictors for POPF were ASA grade 1–2 (OR 2.32, CI 1.32–4.17; *p* = 0.004), operative time ≥ 3 h (OR 2.37, CI 1.35–4.24; *p* = 0.003), and duct size < 3 mm (OR 3.03, CI 1.23–8.33; *p* = 0.023). Independent post-operative laboratory predictors were drain amylase activity ≥ 50μ-kat/L on POD3 (OR 4.35, CI 2.22–4.66; *p* < 0.001) and white blood cell count ≥ 18 × 10^9^/L on POD1 (OR 2.52, CI 1.36–4.66; *p* = 0.003). Neither elevated serum amylase activity on either one of the first 3 days nor CRP levels on POD2 were independent predictors for POPF.

**Fig.1:**
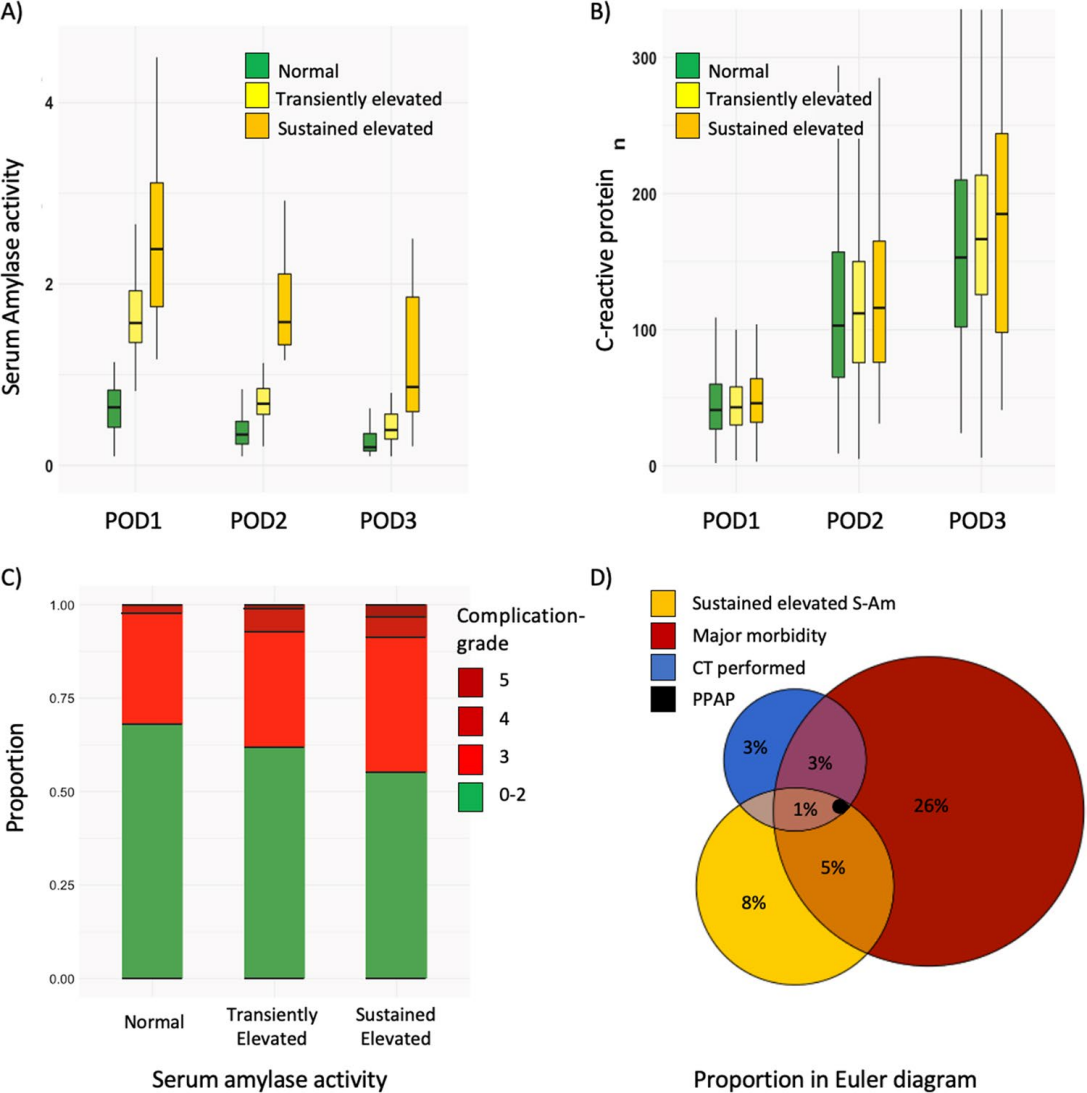
**A**, **B** Boxplots of serum amylase activity and CRP levels on POD1, POD2, and POD3 for the three primary serum amylase activity levels. **C** Bar plot showing the frequency of complications stratified by the same three serum amylase activity levels. **D** An Euler diagram depicting the relationship between serum amylase activity, performed CT, major complications, and PPAP diagnosis

Figure 1 displays boxplots of serum amylase activity and CRP levels on POD1, POD2, and POD3 for the three primary serum amylase activity levels, and a bar plot shows the frequency of complications stratified by the same three serum amylase activity levels. Additionally, an Euler diagram depicting the relationship between serum amylase activity, performed CT, major complications, and PPAP diagnosis was included.

## Discussion

Among the 403 patients who underwent DP at a tertiary center in this study, only one patient met the PPAP criteria — primarily due to the infrequent use of CT scans and the low incidence of findings consistent with acute pancreatitis. The study also found that 46% exhibited elevated serum amylase activity post-operatively. Of those, 40% developed major complications, but there was no significant difference in the rate between patients with transiently and sustained elevated activity. Further, peaked serum amylase activity was, contrary to sustained elevated serum amylase activity, an independent adverse predictor for complications. These findings suggest reconsidering the need for radiology in PPAP diagnosis, and advising clinicians to be vigilant when patients having undergone DP displays a peaked elevated serum amylase activity, even if only transient.

In non-surgical contexts, acute pancreatitis can be diagnosed in 80% of cases using biochemical and clinical criteria. The widely utilized biochemical criterion is a serum amylase activity ≥ 3 times normal.^[15]^

The present study confirmed that elevated serum amylase activity detected as early as POD1 following DP is associated with complications.^[16]^ Patients who displayed peaked serum amylase activity, typically on POD1, were more likely to experience major complications, with the peak activity serving as an independent predictor for such outcomes. While sustained elevation of serum amylase activity was linked to major morbidity almost half of the time, univariable analysis did not show a significant correlation. These results suggest that the level of serum amylase activity is directly associated with patient outcome and that the sustained component may be secondary to situations that trigger an initial peak of serum amylase with trailing POH on subsequent day(s), rather than being the causal driver per se for major complications.

Nevertheless, patients with peaked serum amylase activity, either transiently or sustained elevated, may represent different underlying causes when compared to those with sustained elevation without a peak. Non-ideal intra-operative manipulation of a soft pancreas^[17,18]^ and excessive stitching of the pancreatic remnant,^[19]^ which can lead to alterations in blood supply and local ischemia,^[20]^ are factors that may trigger POH, with or without subsequent major complications. In this study, we observed that both serum amylase conditions were linked to extended extrapancreatic resection and lower ASA grades. Additionally, sustained elevated activity was associated with a pancreatic duct ≥ 3 mm, possibly due to pre-operative conditions leading to partial occlusion of the pancreatic duct. More research is needed to comprehensively understand the initial stages of POH and its association with major complications in patients resected with DP.

The second diagnostic criterion for acute pancreatitis for non-surgical cases is abdominal pain. However, in post-surgical patients who receive adequate pain management, the assessment of abdominal pain cannot be considered reliable.^[21]^ Nevertheless, pain in acute pancreatitis is a manifestation of the emerging pancreatic inflammation triggered by the release of inflammatory mediators.^[22]^ The inflammatory mediator CRP is well-correlated with the extent of the pancreatitis^[15]^ and can predict complications related to POH with cut-off value of 180 mg/L.^[2]^ In the current study, CRP level of ≥ 180 on POD2 was also correlated with complications, but the correlation was even more robust for a CRP level of ≥ 200 on POD3, which also independently predicted adverse outcomes.

Thus, serum amylase activity along with CRP on POD2–3 can be helpful in the diagnosis of significant pancreatitis when carefully evaluated within a clinical context that takes into account symptoms like fever, nausea, vomiting, tachycardia, tachypnea, hypotension, and oliguria. Additional research is necessary to explore the associations outlined above.

Imaging, the third criterion for diagnosing acute pancreatitis in non-surgical settings, is not commonly used,^[23,24]^ and most cases display only subtle changes.^[25,26]^ Likewise, interstitial changes in acute pancreatitis related to surgery may also exhibit discrete findings that may go undetected on early imaging but may be significant enough to jeopardize the closure of the pancreatic remnant.

A recent study assessed the clinical impact of POH on POD1 in relation to acute pancreatitis after DP.^[16]^ CT findings were retrospectively evaluated. Of 641 resected patients, 143 (22%) underwent CT within 2 weeks, and only 10 (7%) had radiologic findings consistent with acute pancreatitis, corresponding to 1.6% of the entire cohort. In the present study, 30 patients (8%) underwent CT within the first week, and only one of those (3%) demonstrated findings consistent with acute pancreatitis. Together with clinical and biochemical criteria, this corresponds to an incidence of PPAP of 0.25% in our cohort.

In order to identify milder forms of inflammation, we clearly need more sensitive diagnostic methods than abdominal CT during the portal venous phase. Imaging should be used for cases requiring diagnostic clarification. Future research should focus on identifying patterns of post-operative serum amylase activity and inflammatory markers in conjunction with clinical parameters to establish risk assessment tools for post-operative complications.

POPF is the leading cause to major morbidity after DP,^[27,28]^ but despite being associated with predictors related to wound healing, exocrine function, and pressure in the sphincter of Oddi,^[29]^ prediction of its occurrence remains unreliable. Acute pancreatitis defined by Connor^[2]^ has been suggested to be an additional factor for development of POPF after DP.^[1]^ Our study found that PPAP and POPF were both associated with ASA and duct diameter, but PPAP alone was not an independent predictor of POPF. This suggests that PPAP may play a mediating or moderating role in the development of POPF and that the definition of inflammation in the pancreatic remnant needs further clarification.

To summarize, it is likely that surgical acute pancreatitis like the non-surgical counterpart is mild to moderate and have a brief course. These cases can be diagnosed based on biochemical and clinical criteria alone, and the suggested laboratory criteria by ISGPS may miss cases with important post-operative complications. Analysis of serum amylase and CRP in a clinical context could suffice for diagnosis in most cases. CT is usually not performed for non-surgical acute pancreatitis or after pancreatic surgery, and when it is performed, it can be diagnostically challenging in the post-operative setting. Criteria more consistent with the Atlanta classification could provide an accurate diagnosis of acute pancreatitis in most cases and serve as important early triage.

There are some important limitations of the present study that need to be considered. Firstly, it was a retrospective study from a single center. Secondly, missing values of serum amylase activity on POD2 may have altered the results. This temporal detail may have implications on the analyses performed.

## Conclusion

The present study found that PPAP after DP is rare and that computed tomography has limited usability for diagnosing PPAP. Using complementary imaging and considering patients with transiently elevated serum amylase activity, especially if peaked, could help identify additional important presentations. Strict adherence to imaging requirements may also lead to unnecessary diagnostics for patients with POH and an expected normal post-operative course. Further research is needed to better understand POH dynamics, refine cut-off levels, and identify clinically accurate predictors for diagnosis.
